# Seed Transmission of Begomoviruses: A Potential Threat for Bitter Gourd Cultivation

**DOI:** 10.3390/plants12061396

**Published:** 2023-03-21

**Authors:** Ravisankar Gomathi Devi, Chinnaraj Jothika, Arjunan Sankari, Sethuraman Lakshmi, Varagur Ganesan Malathi, Perumal Renukadevi

**Affiliations:** 1 Department of Plant Pathology, Tamil Nadu Agricultural University, Coimbatore 641003, Tamil Nadu, India; 2 Department of Vegetable Science, Tamil Nadu Agricultural University, Coimbatore 641003, Tamil Nadu, India; 3 Department of Seed Science and Technology, Tamil Nadu Agricultural University, Coimbatore 641003, Tamil Nadu, India; 4 Retired Scientist, ICAR-IARI, GI, Sree Kumaran Hill Crest Apartment, Coimbatore 641046, Tamil Nadu, India

**Keywords:** *Momordica charantia* L. embryo, grow-out test, microplot, ToLCNDV, BgYMV

## Abstract

Bitter gourd (*Momordica charantia* L.), one of the valued vegetable crops in India, is severely affected by yellow mosaic disease caused by two begomoviruses, tomato leaf curl New Delhi virus (ToLCNDV) and bitter gourd yellow mosaic virus (BgYMV). The symptoms are yellowing, distortion of leaf, puckering, and malformed fruits. Increased incidence of the disease and appearance of symptoms even in young emerging seedling stage were suggestive of seed transmission of the viruses, which was examined in detail. To study the seed transmission, two sources—seeds of elite hybrids H1, H2, H3, H4, and Co1 procured from a seed market; and seeds from infected plants in the farmer’s field were tested. Detection of the virus by DAS-ELISA using polyclonal antibody indicated embryo infection up to 63%, 26%, 20%, and 10% in hybrids H1, H2, H3, and H4, respectively, for market-procured seeds. In PCR analysis with primers specific for ToLCNDV and BgYMV, infection by ToLCNDV was as high as 76% and mixed infection was 24%. In contrast, in seeds derived from field-infected plants, the percentage detection was less. Grow-out tests with market-procured seeds revealed no transmission for BgYMV compared with 5% transmission for ToLCNDV. Whether seed-borne inocula could serve as an inoculum for new infection in a field and further progress of the disease was investigated in a microplot study. The study clearly revealed variation in seed transmission between different sources, lots, cultivars, and viruses. The virus present in symptomatic and asymptomatic plants was easily transmitted by whitefly. In another microplot experiment, the potential of seed-borne virus as inoculum was proved. There was 43.3% initial seed transmission in the microplot, increasing to 70% after release of 60 whiteflies.

## 1. Introduction

Emergence of diseases caused by begomoviruses is the major challenge in improving productivity of food, feed, fiber, and ornamental crops. The genus *Begomovirus*, which belongs to the family *Geminiviridae*, contains nearly 450 virus species affecting a large number of economically important crops. Virus members of this family have a circular single-stranded genome of 2.7–3 kb length, encapsidated by twinned para-icosahedral virion particles (22 × 38 nm). On the basis of genome organization, vector, and host range, the family is divided into 14 genera—*Becurtovirus*, *Begomovirus*, *Capulavirus*, *Curtovirus*, *Eragrovirus*, *Grablovirus*, *Mastrevirus*, *Topocuvirus*, *Turncurtovirus, Citlodavirus*, *Maldovirus*, *Mulcrilevirus*, *Opunvirus*, and *Topilevirus* [1,2,3,4]. Of all the ssDNA viruses, the genus *Begomovirus* infects economically important dicotyledonous hosts, and is transmitted by the whitefly, *Bemisia tabaci*, in a circulative persistent manner. The begomoviruses have either a bipartite (DNA A and DNA B) or monopartite genome (DNA A) encapsidated within geminate particles. The transcription is bidirectional, and genes encoded in virion-sense and complementary-sense strands are separated by an intergenic noncoding region comprising an invariable stem–loop region consisting of the nonanucleotide sequence TAATATTAC representing the origin of replication. DNA A component encodes for pre-coat protein (ORF AV2) and coat protein (ORF AV1, CP), in the virion-sense strand; the complementary strand encodes for replication initiator protein (ORF AC1, Rep), transcription activator protein (ORF AC2, TrAP), replication enhancer protein (ORF AC3, REn), and a symptom determinant (ORF AC4 sd) protein. The DNA B component encodes for nuclear shuttle protein (ORF BV1, NSP) on the viral strand and cell to cell movement protein (ORF BC1, MP) on the complementary strand.

Bitter gourd (*Momordica charantia* L.), also known as bitter melon and belonging to the family *Cucurbitaceae*, is cultivated in tropical regions such as Africa, China, India, Indonesia, and Malaysia. Due to its high medicinal value (antidiabetic), there is high demand for the fruit and seeds. Unfortunately, it is affected by several plant pathogens of which viral diseases caused by whitefly-transmitted begomoviruses are major constraints. In India, bitter gourd has been plagued by a severe yellow mosaic disease in the past decade. The symptoms are yellowing, leaf lamina distortion, puckering, and stunted growth of the plants. The begomovirus species associated with the disease in India are tomato leaf curl New Delhi virus (ToLCNDV) [5,6,7,8] and bitter gourd yellow mosaic virus [9].

The spread of viral diseases over vast distances is mainly due to seed transmission of viruses. True seed transmission is defined as passage of the virus from the embryo of the infected plant to the next generation seedling. Real seed transmission, in contrast to the seed-borne nature of viruses, is exceedingly limited since seed embryos are highly protected against viral invasion. Although seed transmission of the beet curly top and abutilon mosaic viruses had been theorized since 1868 [10], conclusive proof for seed transmission of begomoviruses utilizing virus-specific molecular methods was only presented in 2015 for the sweet potato leaf curl virus (SPLCV) [11]. Transmission from seed to seedling was subsequently demonstrated in about 13 host–virus combinations—tomato yellow leaf curl virus (TYLCV) in soybean and sweet pepper [12,13], mungbean yellow mosaic virus (MYMV) in blackgram [14], tomato leaf curl New Delhi virus (ToLCNDV) in chayote [15] and zucchini squash [16], BgYMV in bitter gourd [9], dolichos yellow mosaic virus (DoYMV) in lablab bean [17], sweet potato symptomless virus I (SPSMV) in sweet potato [18] and pepper leaf curl Indonesia virus in chili capsicum (PepYLCIV) [19], and okra yellow mosaic Mexico virus (OYMMV) [20]. Manivannan et al. [9] recorded seed infectivity by BgYMV up to 79.16% and a transmission rate of 32% in seedlings. In the present communication, results on detailed studies on seed transmission of two begomoviruses ToLCNDV and BgYMV in different bitter gourd hybrids are presented. Evidence is provided to prove the role of seed-borne inoculum in contributing to infection and disease progress in the field.

## 2. Results

### 2.1. Detection of Begomoviruses in Market Seeds

#### 2.1.1. DAS-ELISA

**Figure 1:**
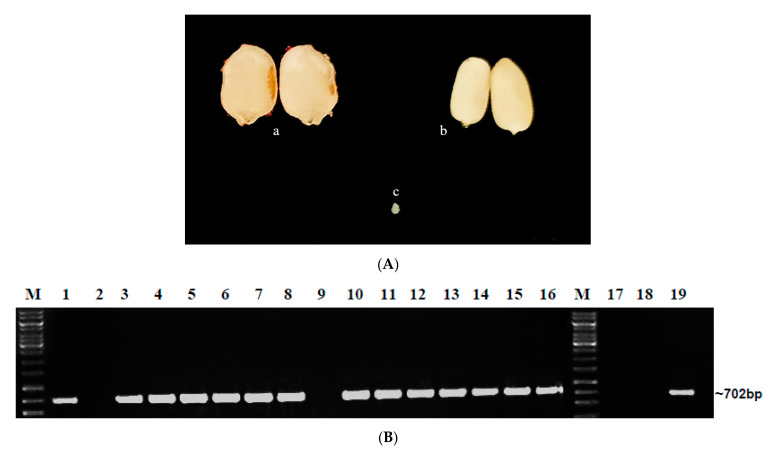
(**A**) Different parts of seeds dissected for DAS-ELISA; a. Seed coat, b. Endosperm. c. Embryo. (**B**) Detection of ToLCNDV in DAS-ELISA-positive embryo samples of H2 seeds from market; Lane-M—1 Kb Ladder, L1–L16—H2 Embryo samples, Lane M—1 Kb Ladder, L17, 18—H2 Embryo samples; L16, 19—PC.

Seeds collected from the market were cleaned, dissected, and used (Figure 1A). The double antibody sandwich–enzyme-linked immunosorbent assay (DAS-ELISA) conducted with ToLCNDV antiserum in market-procured seeds revealed that among four hybrids, the percentage of embryo infection was high in H2 (62.96%) followed by H1 (26.6%), H3 (20%), and H4 (10%). The variety CO1 recorded nil embryo infection (Table 1 and Supplementary Materials Table S1).

#### 2.1.2. PCR Using ToLCNDV and BgYMV Specific Primers

The embryo samples that were positive for DAS-ELISA were further analyzed for the presence of the specific begomoviruses ToLCNDV and BgYMV through PCR. In H1 seeds, out of eight embryos positive in DAS-ELISA, four were positive for ToLCNDV alone and two were positive for both BgYMV and ToLCNDV, the other two samples were PCR-negative. In H2 seeds, out of 17 embryos positive in DAS-ELISA, 13 were positive for ToLCNDV alone (Figure 1B), and four were positive for both BgYMV and ToLCNDV. In H3 seeds, out of six embryos positive in DAS-ELISA, all the six embryos were positive only for ToLCNDV. In H4 seeds, out of three embryos positive in DAS-ELISA, all the three embryos were positive only for ToLCNDV. Among all the hybrids, H1 and H2 showed the presence of both begomoviruses BgYMV and ToLCNDV (Table 2).

### 2.2. Detection of Begomoviruses in Seeds Collected from Infected Bitter Gourd Plants in the Field

#### 2.2.1. DAS-ELISA

**Figure 2:**
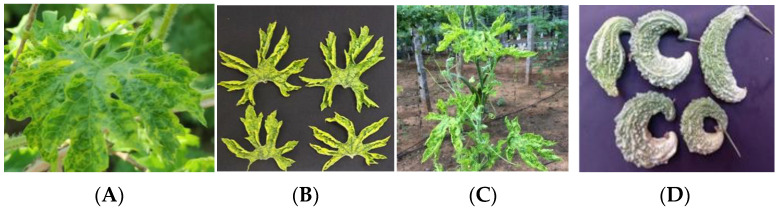
(**A**) Severe mosaic—BgYMV alone (**B**) Severe Mosaic and upward rolling—ToLCNDV alone (**C**) Mild mosaic and upward curling—ToLCNDV and BgYMV (**D**) Malformed fruits from plant with both viruses.

The plants showing clear symptoms of yellowing, mosaic, leaf deformities, and bushiness were tagged and malformed fruits were collected (Figure 2A–D). The seeds were dissected and subjected to ELISA. The results of DAS-ELISA conducted with ToLCNDV antiserum analysis in seed parts from infected plants revealed that the percentage of embryo infection was high in H2 (33.3%) followed by H1 (26.6%) and H3 (20%) (Table 1 and Table S1).

#### 2.2.2. PCR Using BgYMV- and ToLCNDV-Specific Primers

**Figure 3:**
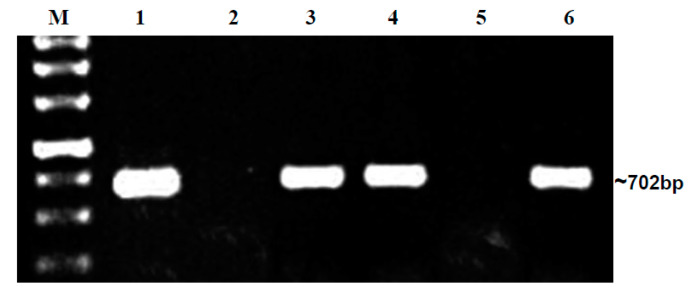
Detection of ToLCNDV in DAS-ELISA-positive embryo samples of H2 seeds from field-collected seeds. M—1 Kb Ladder, L1–L5—H2 Embryo samples; L6—PC.

The embryo samples that tested positive for DAS-ELISA were put through PCR with specific primers for BgYMV and ToLCNDV. In Hybrid 1, out of four embryos positive in DAS-ELISA, three were positive for ToLCNDV alone, and one was positive for both BgYMV and ToLCNDV. In Hybrid 2, out of five embryos positive in DAS-ELISA, three were positive for ToLCNDV alone (Figure 3), and two were positive for both BgYMV and ToLCNDV. In Hybrid 3, all the three embryos positive in DAS-ELISA were also positive only for ToLCNDV alone. Similar to market seeds, seeds of H1 and H2 collected from begomovirus-infected field also revealed the presence of both the begomoviruses. The embryo samples were sequenced, and sequences are available for ToLCNDV (OP096433) and BgYMV (OP096428) in the NCBI database. From our repeat PCR analysis, we infer that both ToLCNDV and BgYMV are present in embryos of bitter gourd plants cultivated in Tamil Nadu (Table 2).

### 2.3. Assessing Seed Transmission by Grow-Out Test for Market Seeds in Glasshouse

**Figure 4:**
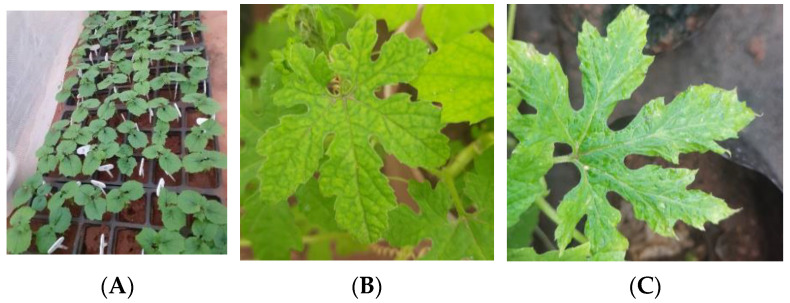
(**A**) Grow-out test with market and infected field seeds in glasshouse (**B**) Yellowing symptom (**C**) Mottling symptom.

Grow-out studies for market collected H1, H2, and H3 hybrids were carried out in an insect-proof glasshouse, using 100 seeds for each hybrid to evaluate seed transmission (Figure 4A). Symptom expression was recorded. The most common symptoms observed were yellowing, puckering, mosaic mottling, and dark green area in lamina alternating with light green in 10 plants out of 300 plants (Figure 4B,C). However due to diffused light, exact bright symptoms observed in the field were not expressed. The leaves of all the 100 plants of H1, H2, and H3 were tested through PCR for BgYMV and ToLCNDV as it will give the specific identity of the virus. Results of PCR analysis revealed that there was no seed transmission of BgYMV in H1, H2, or H3; about 5% seed transmission of ToLCNDV was observed in H1 and H2 and 3% seed transmission in H3. The PCR products from leaf samples were sequenced and they are available in the NCBI database under the following accession numbers—ToLCNDV ON974876, OP06432 (H1), and OP096430 and OP096431 (H2) (Table 3).

### 2.4. Detection of Begomovirus in Grow-Out Test Plants in Microplot Study I

**Figure 5:**
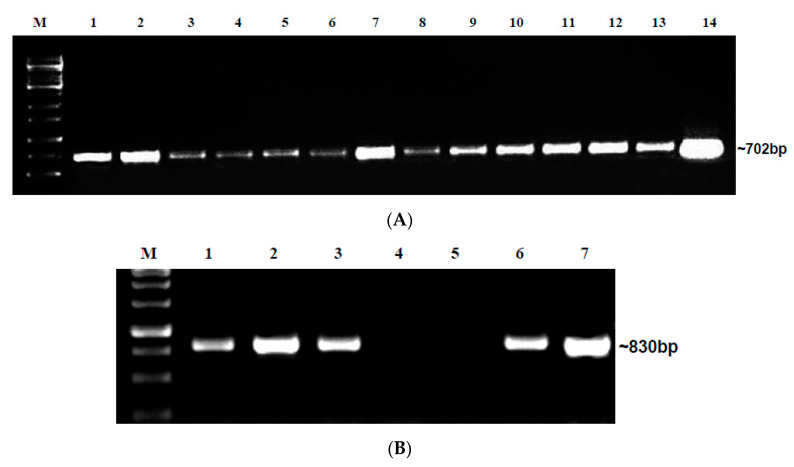
(**A**) Detection of ToLCNDV in grow-out test leaf samples from microplot I. M—1 Kb ladder; L1 to 13 grow-out test leaf samples—ToLCNDV, L14—PC. (**B**) Detection of BgYMV in grow-out test plants in microplot I. M—1 Kb ladder; L1 to 6—Grow-out test leaf samples-BgYMV, L7—PC.

The microplot with a completely closed insect-proof net was divided into two halves, and seeds of H1 collected from the market and seeds of the same hybrid collected from the virus-infected field in Kallimedu were sown in ridges and furrows (Figure S1a–c). Observations were recorded periodically and from 45 days after sowing, leaves were collected, and PCR analysis was carried out using BgYMV- and ToLCNDV-specific primers. Out of 170 plants, 16 expressed symptoms such as light and dark green mottling and severe mosaic (Figure 5A,B). Severe symptoms of yellowing and puckering were not observed due to the diffusion of light through the net. In test plants from market seed, 4.8% plants were symptomatic while 38.4% were asymptomatic and tested positive for ToLCNDV alone. Only 3.2% of plants were symptomatic and 0.8% asymptomatic, while testing positive for both viruses (Table 4 and Table S2). In test plants from infected field seeds, only 2.2% were symptomatic and positive for ToLCNDV alone, while 6.6% plants were symptomatic and 2.2% were asymptomatic and positive for both viruses. It could be inferred that ToLCNDV alone recorded the highest seed transmission of 43.2%, in contrast to infection by both viruses (4%) (Table 4 and Table S2, Figures S2a–g and S3a–f). The PCR products from the leaf samples were sequenced and they are available with the following accession numbers in the NCBI data base—ToLCNDV (ON950085), (OP096429), and BgYMV (OP096427).

### 2.5. Tracking of Begomoviruses in Floral and Fruit Parts of Grow-Out Test Plants in Microplot through PCR

#### Grow-Out Test Plants Derived from Market and Field-Collected Seeds

Tracking of ToLCNDV and BgYMV in floral and fruit parts of grow-out test plants in microplot derived from the market or the infected field revealed a large degree of variation in the distribution of viruses in floral parts. For ToLCNDV, virus was detected in sepals, petals, stamens, and fruit parts of both asymptomatic and symptomatic plants (Figure S4a,b). However, for BgYMV, flowers and fruits collected from symptomatic grow-out test plants from market seeds were found not to harbor virus; but in test plants from the infected field, flowers and fruits of asymptomatic plants and flowers of symptomatic plants did not contain virus; however, the fruit parts and whole seeds were positive for BgYMV (Table S3).

### 2.6. Transmissibility of Begomovirus from Grow-Out Test Plants of Microplot by Whiteflies

**Figure 6:**
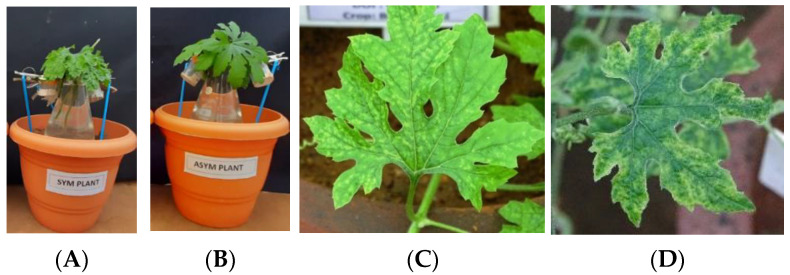
Whitefly transmission of ToLCNDV from microplot grow-out test plants. (**A**) Whiteflies feeding on symptomatic leaves −24 h (AAP); (**B**) Whiteflies feeding on Asymptomatic leaves −24 h (AAP); (**C**) Symptom expressed in plants transmitted from symptomatic plants (**D**) Symptom expressed in plants transmitted from asymptomatic plants.

Following PCR analysis results of microplot grow-out test plants, the potential of grow-out seedlings to serve as inoculum for whitefly (*B. tabaci*) transmission was assessed. The whiteflies were initially maintained in an insect-proof glasshouse on brinjal plants. Crude extract from six whiteflies were subjected to PCR amplification using mtCOI primers, which yielded the expected amplicon of 800 bp on agarose gel electrophoresis. On the basis of sequence identity, the whitefly population maintained for this present study was identified as Asia I genotype (Accession No. OP049919). Among ToLCNDV and BgYMV, ToLCNDV was predominantly present compared to BgYMV in grow-out test plants of both seeds collected from the market and from the virus-infected field. Hence, transmissibility of ToLCNDV was assessed by whitefly transmission test only for ToLCNDV. Leaves from both symptomatic and asymptomatic plants that were positive only for ToLCNDV were used as the source for whitefly-mediated transmission studies. The PCR-negative plants in the microplot study were used as a negative control. Twenty whiteflies were allowed to feed on each of the symptomatic and asymptomatic plants (Figure 6A,B). The results revealed that the virus was transmitted from both asymptomatic and symptomatic plants. Symptom expression was observed 20 days after inoculation. Out of 15 inoculated plants, three expressed symptoms of crinkling and severe puckering; in inoculation with symptomatic plants as source, out of six plants, two showed symptoms of mosaic and mild puckering (Figure 6C,D). In the case of whiteflies fed on healthy leaves, plants did not show any symptoms. Total DNA was isolated from three whiteflies after 12 h of IAP from each category and analyzed through PCR using ToLCNDV-specific primers, which gave the expected amplicon of 702 bp for ToLCNDV. Total DNA was isolated from all leaves for each category and analyzed through PCR using ToLCNDV-specific primers. In the PCR analysis, the whitefly transmission for symptomatic plants revealed that out of 15 plants, two were positive for ToLCNDV with an amplicon of 702 bp, whereas for asymptomatic plants, out of 15 plants, three were positive for ToLCNDV (Figure S5a,b). In both cases, only symptomatic plants were positive. All six healthy plants were found to be PCR-negative for ToLCNDV (Table 5).

### 2.7. Seed as Potential Source of Inoculum for Active Spread of the Disease by Whitefly (Microplot Study II)

In microplot study II, how far the seed serves as a source of inoculum for whitefly transmission was examined. Out of 150 H1 hybrid plants, 22 were BgYMV-positive, 43 for ToLCNDV, and six were positive for both the viruses before the release of whiteflies. After the release (15 days after release) of whiteflies, there was increase of 14 (total 36) plants positive for BgYMV and 29 (72) plants for ToLCNDV. The seed transmission was 43.33% to begin with, whereas 15 days after the release of 60 whiteflies, there was an increase of 28.66% spread in the microplot. Therefore, out of 150 plants, 108 plants harbored the virus, which is about 72% infection (Table 6). The results show that seed is a strong source of inoculum for whiteflies for the spread and enhancement of the severity of the disease. In the begomovirus detection study in whiteflies, out of 15, two were positive for BgYMV alone, five for ToLCNDV alone, and three were positive for both the viruses on PCR (Table 6).

## 3. Discussion

The passage of the virus from diseased plants to their progeny is referred to as seed transmission. Seed transmission has been recorded in about 18% of plant viruses with RNA as their genome [21,22]. They belong to the families *Potyviridae*, *Tobamoviridae*, *Comoviridae*, *Flexiviridae*, *Partitiviridae*, and viroids. Among DNA viruses, the seed-borne nature of cocoa swollen shoot virus was the only case reported earlier [23]. Seed-borne and seed transmission in two geminiviruses, abutilon mosaic virus (AbMV) [24] and beet curly top virus (BCTV) [25], was suspected but lacked evidence. Kim et al. [11] revealed the presence of SPLCV in seeds. Since then, seed-borne and seed transmission of about 13 geminiviruses have been described [10]. In the present investigation, results of seed-borne and seed transmission of two begomoviruses—BgYMV and ToLCNDV—in bitter gourd by molecular detection and grow-out tests are provided. The importance of the results is discussed in the context of seed-borne virus as inoculum and increased begomovirus infection in vegetable crops.

The first aim of the study was to evaluate the extent to which bitter gourd seeds carry begomoviruses. Three popular hybrids of bitter gourd in Tamil Nadu were chosen for the study. In addition, the variety CO1 and the recently released hybrid H4 were also investigated. Preliminary sequencing analysis of the PCR product obtained with virus-specific primers indicated the presence of only BgYMV and ToLCNDV in all the infected bitter gourd plants. Therefore, further studies on seed transmission in bitter gourd focused only on these two viruses. To assess the extent of virus infection, two sources of seeds were analyzed. One set of seeds was procured from the seed market at Coimbatore and the other set was seeds of the same cultivars collected from infected plants in the field. It was assumed that market seeds might be made up of a random assortment of seeds collected from infected and non-infected fields, and that the likelihood of detection might be low. It was hypothesized that in the case of seeds from the infected field, detection level of virus will be much more reliable. In DAS-ELISA tests, the hybrid H2 recorded the highest positive samples in embryo (17) followed by H1 with embryo (8). The percentage of embryo infection was high in H2 (62.96%) followed by H1 (26.6%), H3 (20%) and H4 (10%). For these tests, three embryos were pooled and used for PCR analysis. Even assuming a minimum infection of one embryo among the three, the percentage embryo infection will be 20% for H1, 8.8% for H2, and 6.6% for H3. This magnitude of embryo infection is very high and perhaps explains the emerging disease scenario in bitter gourd. However, in CO1 other seed parts were positive; the embryo did not contain the virus. Seed part analysis revealed that the hybrid H2 recorded the highest number of positive samples for the seed coat.

It is evident that the virus is present as fully assembled particles based on the positive DAS-ELISA results. MYMV-infected seeds were previously reported to significantly contain geminate particles [14]. They also showed the presence of virion by confocal microscopy using coat protein-specific fluorescent-labeled antibody. Only the present study and the investigations on MYMV [14], BgYMV [9] and DoYMV [17] have used DAS-ELISA to detect virion protein contrasting to other geminivirus studies.

When DAS-ELISA-positive embryo samples were tested through PCR-specific primers, ToLCNDV was detected in three embryos in H1, H2, and H3, and BgYMV along with ToLCNDV in one embryo (H1) and two embryos (H2), confirming the earlier observation that ToLCNDV is the most predominant virus. Contrary to our expectation, market seeds were more infected than seeds collected from the field, indicating variation in the amount of virus inoculum in seeds. Conflicting results obtained for the same cultivar from two different sources in our study are similar to previous results [11,26,27,28,29]. This discrepancy in the virus detection between different sources, different batches of the same cultivar, and different cultivars of the same host may explain the ambiguity recorded in seed transmission of some of the begomoviruses.

The migration of the virus within the seed tissue appears to vary from sample to sample and from variety to variety as revealed by results for the seed coat, endosperm, and embryo. Distribution of the virus within the seed tissue was studied by various workers, which again shows vast differences. Most of the 11 geminiviruses that have been investigated have demonstrated the presence of virus in whole seeds, as in the case of SPLCV in sweet potato [15], MYMV in blackgram [14], TYLCV in soybean and sweet pepper [12,13], BgYMV in bitter gourd [9], SPSMV-1 in sweet potato [18], and pepper yellow leaf curl Indonesia virus (PepYLCIV) in chili [19].

There were also differences between symptomatic and asymptomatic plants as regards ToLCNDV and BgYMV. Kim et al. [11] showed the presence of SPLCV in the petals, stamens, pistils, and seeds of two plants of sweet potato cultivar Mokpo-60. Kil et al. [27] detected 20–100% of TYLCV in the floral tissue of tomato plants. Suruthi et al. [17] detected DoYMV in the sepals, petals, and infected mature pods. Manivannan et al. [9] detected BgYMV in petals, sepals, and stamen. Chang et al. [30] detected ToLCNDV–CB and ToLCNDV–OM in cross-pollinated cucumber progenies at a rate higher than 70%. Their results showed that ToLCNDV, tomato leaf curl Taiwan virus (ToLCTV), and tomato yellow leaf curl Thailand virus (TYLCTHV) can be transmitted via seeds or pollens of cucumber and tomato plants.

Virus in embryo has been revealed in SPLCV in sweet potato [11], MYMV in blackgram [14], TYLCV in tomato and sweet pepper [13,27], DoYMV in Lablab bean [17], ToLCNDV in chayote [15], zucchini squash [16], BgYMV in bitter gourd [9], and SPSMV-1 in sweet potato [18].

The grow-out experiments conducted in insect-proof glasshouses provide evidence for seed transmission. One hundred seeds of each hybrid were sown, and the expression of symptoms as well as the PCR detection of ToLCNDV and BgYMV were examined with 3.3% of all plants as a whole expressing symptoms. Considering all hybrids together, surprisingly, BgYMV was not found in any of the 100 plants from any of the three hybrids. ToLCNDV, however, was found in 13 different plants. Grow-out tests have been performed for all the 13 geminiviruses in which seed transmission has been recorded [10]. In the microplot study, though seed transmission was as high as 43.2% for ToLCNDV, the percentage of asymptomatic plants was also high (38.4%). Despite having a higher virus concentration, the plants in a substantial proportion of host–virus combinations do not show symptoms. Absence of symptom expression can be attributed to the lack of a sufficient amount of inoculum and favorable environmental factors needed for symptom expression. However, it can also be due to the recovery phenomenon frequently met with begomovirus infection [31], due to effective RNAi defense by the hosts.

The existence of particle protein inside the seed raises critical questions about the virus entry and internal movement mechanisms. Our earlier understanding of seed anatomy was that angiosperm seeds have vascular distribution only up to the hilum, and plasmodesmatal connections are absent between the cells. Geminiviruses are normally confined to phloem tissues. Due to the aforementioned factors, the entry and spread of the geminivirus in the seed were thought to be almost impossible. In the context of increasing evidence for seed transmission of geminiviruses, Renukadevi et al. [10] suggested a model based on the hypothesis proposed by Wang and Maule [32,33,34]. Wang and Maule showed the presence of a column of suspensor cells that ensures nutrient supply to the embryo. They proposed that virus transport could occur through these columnar suspensor cells. The suspensor cells are short-lived, and so only a short window period is available for the virus to move from paternal to embryonic tissue. In order for the virus to enter the embryo through columnar cells, Wang and Maule [34] and Renukadevi et al. [10] suggest that the virus’ concentration in the endosperm must be high and continuous accumulation of the virus for the appropriate period of time is required. As demonstrated by Wang and Maule [34] for permissive and non-permissive hosts in pea seed-borne mosaic virus (PSBMV), hosts may differ in this feature. In the present investigation, the concentration of the virus in the endosperm was higher in H1 and H3 hybrids. The embryo of the CO1 variety was totally negative, showing absence of movement of virus from endosperm to embryo. This difference was observed among genotypes, and seed transmission may depend on genotypes, during which time gating and abundant viral concentration in the endosperm might decide seed transmission. Transport proteins are considered to be involved in symplast/apoplast docking of nutrients in seed tissues [35]. The interaction of these proteins with the viral protein might shed further light on viral movement into the embryonic tissue.

**Figure 7:**
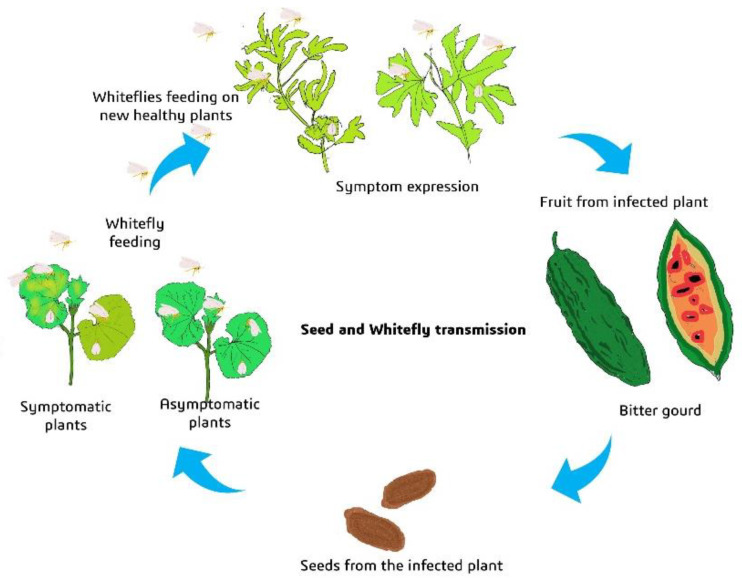
Seed as a potential source of inoculum for the begomoviruses.

Pagán [21] considered seed transmission as an important source of primary inoculum. The potential of an embryo-borne virus to serve as inoculum for initiation of infection and further spread of the virus was proved in the microplot experiments. The initial infection was 43% due to seed transmission, which increased up to 72% after release of whiteflies. We also showed whiteflies acquiring and transmitting viruses from both symptomatic and asymptomatic plants. Kil et al. [27] demonstrated whitefly transmission from seedlings raised from TYLCV-infected seeds in an insect-rearing tent. After eight weeks of co-cultivation, three plants produced typical TYLCV symptoms. The possibility of whitefly transmission from symptomatic and asymptomatic plants raised from virus-borne embryos and spread of this inoculum have been established for the first time in this study (Figure 7).

Overall, this study established that seed transmission of ToLCNDV and BgYMV is a threat to be reckoned with. Subsequently, seed treatment for mitigation of initial inoculum and phytosanitary regulatory guidelines need to be developed to protect the cultivation of bitter gourd.

## 4. Materials and Methods

### 4.1. Detection of Begomoviruses in Different Seed Parts through DAS-ELISA and PCR

#### 4.1.1. Collection of Market Seeds

Bitter gourd seeds of four major hybrids H1, H2, H3, and H4 (to avoid infringement of privacy, trade names are not provided) were collected from the seed market at Coimbatore. Seeds of the bitter gourd variety CO1 were collected from the Department of Vegetable Sciences, HC&RI, Tamil Nadu Agricultural University, Coimbatore. Among the seeds collected, H1 is predominantly grown by farmers, followed by H2, whereas H3 is grown only in the Pollachi region of Coimbatore district.

#### 4.1.2. Collection of Seeds from Infected Fields

Fruits of three major hybrids, that is H1, H2, and H3, of bitter gourd were collected from three virus-infected fields of Coimbatore district—Kallimedu (KM), Udumalaipettai (UP), and Alangidavu (AG).The plants showed severe symptoms of mosaic, yellowing, leaf inward rolling, stunting, and bushiness. Plants expressing symptoms were tagged in the initial growth stage, and fruits were collected from those plants. Seeds were separated from fruits and air-dried for further analysis.

#### 4.1.3. DAS-ELISA

The seed coat, endosperm, and embryo of bitter gourd seeds collected either from the seed market or from infected plants in the field were dissected. The different seed parts of all four hybrids and the variety Co1 were tested for the presence of the virus. For each hybrid and the variety, 30 seeds were tested. Fifteen samples of seeds of three hybrids collected from infected fields were used for detection of begomoviruses through DAS-ELISA using ToLCNDV antiserum. Embryos of three seeds constituted one embryo sample. Two biological replications for each seed part and flower part were tested in ELISA (two wells each). Embryo samples positive in ELISA tests were subjected to PCR, for the detection of the specific begomoviruses ToLCNDV and BgYMV.

DAS-ELISA was conducted following the manufacturer’s protocol (DSMZ, AS-1109). The healthy bitter gourd plant grown in an insect-proof glasshouse, which tested negative for begomovirus, was used as negative control and symptomatic leaves collected from the field were used as positive control. The experiment was conducted in Nunc-Immuno 96-well microtiter plates (Sigma-Aldrich), with ToLCNDV DAS-ELISA kit from DSMZ Germany (AS-1109). The seed samples were homogenized thoroughly in a sterile pestle and mortar with general extraction buffer (0.05 M tris containing 0.06 M sodium sulfite) at 1 mL/g of sample. The extract was allowed to settle for 10 min and the supernatant was transferred to a new microcentrifuge tube. ToLCNDV IgG was used at 1:1000 dilution for coating and enzyme conjugate of ToLCNDV antibody was applied in 1:500 dilution. One hundred microliters of *p*-nitro phenyl phosphate (*p*-NPP) substrate (Sigma Aldrich, USA) (5 mg/5 mL) was added to each well and incubated in the dark to promote color development. The optical density (OD) at *A*_405_ nm was measured 1 h after the addition of the substrate in a microplate reader (SpectraMax i3). To confirm the presence or absence of virus, readings were taken a second time 18 h after substrate addition. The mean value of the absorbance of two wells of two replications was taken to represent the value of one sample. An absorbance value twice higher than the values obtained in the healthy control was considered as positive for begomoviruses. Appropriate statistical analysis was carried out with absorbance values.

#### 4.1.4. PCR Using BgYMV and ToLCNDV Primers

The embryo sample extracts that were positive in ELISA were subjected to PCR using BgYMV and ToLCNDV primers. Total DNA was extracted from embryo samples used in ELISA using the GEM-CTAB protocol [36]. Finally, a total nucleic acid pellet was suspended in 50 µL of nuclease-free water. The quality of genomic DNA was checked on 1% agarose gel and stored at −20 °C for further use. A set of specific primers for BgYMV and ToLCNDV was used to detect the presence of the virus through PCR analysis. The primers pair BgYMVF 5′-TCATCAATGACGTTGTAC-3′ and BgYMVR 5′-GAGCTTTTGAGTGCACCCG-3′ specific for the BgYMV Rep region spanning nucleotide coordinates (1798–2596) with an expected amplicon size of 830 bp [9] and Bm794F (492–509) 5′-CCTTGTAAGGTGCAGTCC-3′, Bm795R (1194–1175) 5′-AACCCAGGTCCTTAAGTACC-3′ specific for the AV1 and AC3 regions, respectively, of ToLCNDV with an expected amplicon size of 702 bp [37] were used for detection. PCR was performed in 25 μL reaction mixture containing 12.5 μL of 2× SmART Prime master mix, (readymade mix of Taq polymerase, dNTPs, and PCR Buffer; Genei Laboratories Pvt. Ltd., Karnataka, India, cat. No. #280311), 5 μL of template DNA, 2 μL of each forward and reverse primers (100 ng/µL each). The PCR mix volume was made up to 25 µL by adding sterile distilled water and incubated at the following temperature cycle for successful amplification. For BgYMV, initial denaturation of DNA at 94 °C for 2 min was followed by 35 cycles of denaturation 94 °C for 50 s, annealing at 52 °C for 45 s, extension at 72 °C for 1.30 min, and final extension at 72 °C for 15 min. For ToLCNDV, initial denaturation of DNA at 94 °C for 3 min followed by 35 cycles of denaturation 94 °C for 30 s, annealing at 56 °C for 45 s, extension at 72 °C for 1 min, and final extension at 72 °C for 10 min. Finally, the PCR products were visualized by 1% agarose gel electrophoresis for 1 h at 80 V. The DNA extracted from bitter gourd plants was initially tested for the presence of ToLCNDV or BgYMV and served as a positive control in subsequent tests. The PCR products were further sequenced at Syngenome PVT LTD.

### 4.2. Grow-Out Test for Seed Transmission

Hybrids H1, H2, and H3 were purchased from the seed market and sown in plastic pots and maintained in insect-proof cages in the glasshouse. The seeds were soaked overnight in sterile water containing 0.2% Tween 20 as decontaminant. For each hybrid, 100 seeds were sown. Periodically, grow-out test plants were monitored for symptom expression and the days taken for symptom expressions were recorded. Leaf samples from all the plants (both symptomatic and asymptomatic) were collected and analyzed for the presence of begomoviruses by PCR using specific primers for BgYMV and ToLCNDV.

### 4.3. Detection of Begomovirus in Grow-Out Test Plants in Microplot

A microplot of 32 m length, 13 feet height, and a width of 12 m dimension was prepared and covered on all sides with an insect-proof net (40 mesh) on all sides. One hundred twenty-five seeds of H1 collected from the market and 44 seeds of the same hybrid collected from virus-infected field of KM were sown separately in two halves of the microplot. Normal cultivation practices were followed without any plant protection applications. Plants were monitored regularly for symptom expression. Plants showing symptoms were tagged with a red label. At 45 days after sowing, 125 samples from plants grown from seeds collected from the market and 44 samples from plants grown from seeds collected from the virus-infected field were subjected to PCR analysis with specific BgYMV and ToLCNDV primers. Based on PCR results, plants were grouped into three categories—symptomatic plants, asymptomatic plants, and virus-free healthy plants.

### 4.4. Tracking of Begomoviruses in Floral and Fruit Parts in Microplot

Flowers and fruits of infected plants were collected from both symptomatic and asymptomatic plants from both market seeds and seeds from infected field plants and from healthy plants. Three flowers and three fruits each from symptomatic, asymptomatic, and healthy grow-out test plants of field-collected seeds and market seeds of the microplot were used for tracking begomoviruses. Flowers were separated into sepals, petals, and stamens, and fruit parts were separated into outer rind, inner rind, and seed. DNA was isolated from floral and fruit tissue, and PCR and gel electrophoresis were conducted as described earlier to check for the presence of begomoviruses. Three biological replications for each part were analyzed using BgYMV- and ToLCNDV-specific primers.

### 4.5. Transmissibility of Viral Inoculum from Grow-Out Test Plants of Microplot

#### 4.5.1. Establishment of Whitefly Culture

Egg masses of whiteflies from bitter gourd plants were collected and placed on brinjal plants maintained inside the insect-proof rearing chamber in the PL-480 glasshouse for multiplication. For whitefly genotype identification, the lysis method was followed to extract the DNA from whiteflies [38]. The primers for mitochondrial cytochrome oxidase subunit 1 (mtCO1) forward 5′-TTGATTTTTTGGTCATCCAGAA-3′ and reverse 5′-TCCAATGCACTAATCTGCCAT-3′ [39] were used to identify the genotype of whitefly through PCR. The genotype established was determined based on a 3.5% divergence in the chosen mtCOI sequence when compared to other cryptic species.

#### 4.5.2. Whitefly Transmission

Identified whiteflies were utilized in transmission tests with 24 h of AAP and 12 h of IAP [9]. Whitefly transmission was performed only for ToLCNDV-infected plants as it is the most predominant virus. Twenty whiteflies per clip cage were allowed to feed on bitter gourd (H1) leaves (three clip cages per plant) of symptomatic, asymptomatic, and healthy leaves taken from grow-out test plants of the microplot to acquire the virus with 24 h of AAP. The viruliferous whiteflies and aviruliferous whiteflies with 24 h of AAP were carefully removed from the infected source and allowed to feed on 15 healthy 20-day-old bitter gourd plants for each category inside the insect-proof glass chamber to inoculate the virus with 12 h of IAP. Five whiteflies from each category were taken for detection of ToLCNDV and the remaining whiteflies were killed by spraying systemic insecticide (Imidacloprid 200SL @ 0.4 mL/L). Finally, plants were maintained in cages with an insect-proof net for symptom expression. The leaves for transmission study were collected at 20 days post-inoculation and were subjected to PCR analysis with ToLCNDV-specific primers.

### 4.6. Seed as Potential Source of Inoculum for Whitefly (Microplot Study II)

In the same microplot with the insect-proof net, after a 3-month period, ridges and furrows were formed. One hundred fifty H1 hybrid seeds were sown, and standard cultivation practices were followed. Thirty-five days after sowing, leaf samples were collected from all the 150 plants and checked for both ToLCNDV and BgYMV through PCR using specific primers. On Day 45, 60 whiteflies (Asia I genotype) from pure culture were released into the microplot and the progress in disease incidence was observed. On Day 60, leaf samples from all the plants were collected and checked for both ToLCNDV and BgYMV through PCR using specific primers. On the same day, 15 whiteflies were also collected from the microplot for the detection of both begomoviruses.

## Figures and Tables

**Table 1:** Summary of DAS-ELISA results for different bitter gourd seeds.

Sample. No	Hybrid/Variety	Percentage of Embryo Infection (%)
**Hybrid/variety seeds collected from market**		
1.	H1	26.6
2.	H2	62.96
3.	H3	20.00
4.	H4	10.00
5.	Variety Co1	0.00
**Hybrid seeds collected from begomoviruses-infected plant from field**		
6.	H1	26.6
7.	H2	33.33
8.	H3	20.0

Computed on the basis of embryo infection.

**Table 2:** Detection of begomoviruses in ELISA-positive embryo samples of seeds through PCR using specific primers.

Sample. No	Hybrid/Variety	No of Samples Positive/Total No of Samples	
		ToLCNDV Alone	Both BgYMV and ToLCNDV
Hybrid seeds from market			
1.	H1	4/8	2/8
2.	H2	13/17	4/17
3.	H3	6/6	-
4.	H4	3/3	-
Hybrid seeds collected from begomoviruses-infected field			
5.	H1	3/4	1/4
6.	H2	3/5	2/5
7.	H3	3/3	-

- Not detected.

**Table 3:** Detection of begomoviruses in bitter gourd grow-out test plants in glasshouse through PCR.

Sample. No	Hybrid	No of Samples Positive/Total No of Samples Tested		No of Symptomatic Plants	No of Asymptomatic Plants	Percentage of Seed Transmission (%)
		BgYMV	ToLCNDV			
1	H1	0/100	5/100	4/5	1/5	5.0
2	H2	0/100	5/100	3/5	2/5	5.0
3	H3	0/100	3/100	3/3	0/3	3.0

**Table 4:** Detection of begomoviruses in bitter gourd grow-out test plants (leaf samples) in microplot I.

Detection of Begomoviruses Using PCR with Specific Primers (170 Plants)					Percentage of Seed Transmission of Both Combined (%)
ToLCNDV Alone Detected (%)		Percentage of Total Seed Transmission of ToLCNDV Alone (%)	Both BgYMV and ToLCNDV Detected (%)		
Symptomatic Plants	Asymptomatic Plants		Symptomatic Plants	Asymptomatic Plants	
**Grow-out test plants (H1) of seeds collected from market**					
4.8	38.4	43.2	3.2	0.8	4.0
**Grow-out test plants (H1) of seeds derived from infected fields**					
2.2	0.0	2.2	6.6	2.2	8.8

**Table 5:** Transmission of the begomovirus (ToLCNDV) to bitter gourd seedlings using *Bemisia tabaci*.

Inoculum Source (Microplot Study)	Acquisition Access Period (AAP)	Inoculation Feeding Period (IAP)	No of Plants Showing Symptoms/No of Plants Used for Transmission	Days Taken to Express Symptoms	Type of Symptoms	PCR Reaction
**Symptomatic plants raised from market seeds**						
1	24 h	12 h	2/15	20	Mosaic and mottling	+
**Asymptomatic plants raised from market seeds**						
2	24 h	12 h	3/15	20	Puckering, crinkling and mottling	+
**Healthy plants**						
3	24 h	12 h	0/15	Nil	No symptom	−

+ PCR positive; − PCR negative.

**Table 6:** Extent of spread of begomoviruses from seedling borne inoculum through the vector *B. tabaci* in microplot II.

Particulars	No of Samples Positive/No of Samples Tested in PCR BgYMV	No of Samples Positive/No of Samples Tested in PCR ToLCNDV	Extent of Transmission (%)
Before releasing of whitefly in microplot	22/150	43/150	43.33
After releasing whitefly in microplot	+14/150	+29/150	+28.66
Total	36/150	72/150	72.0
Begomovirus detected in whitefly DNA sample	2/15	5/15	

+ Additional infection.

## Data Availability

The data is given in the supplementary link of this journal only.

## Supplementary Materials

The following supporting information can be downloaded at: https://www.mdpi.com/article/10.3390/plants12061396/s1, Table S1: Summary of DAS–ELISA results for different bitter gourd hybrid seeds /Variety collected from market and infected field; Table S2: Detection of begomoviruses in bitter gourd grow-out test plants (leaf samples) in microplot study; Table S3: Tracking of begomoviruses in floral and fruit parts of microplot plants by PCR analysis; Figure S1: (a). Begomovirus infection of H1 seeds collected from market and infected field in microplot I. (b). Mosaic. (c) Green mottling; Figure S2: (a–g) Agarose gel electrophoresis of PCR products of grow-out test plants of H1 (market seeds) in microplot using ToLCNDV primer; Figure S3: (a–f) Agarose gel electrophoresis of PCR products of grow-out test plants of H1 (market seeds) in microplot using BgYMV prime; Figure S4: (a,b) Tracking of begomoviruses in floral and fruit parts of grow out test plants (H1) of microplot study; Figure S5: (a,b). Whitefly transmission of ToLCNDV from microplot grow-out test plants.
