# Supplementary material for: Seed Transmission of Begomoviruses: A Potential Threat for Bitter Gourd Cultivation
Source: Plants (Basel). 2023 Mar 21;12(6):1396. doi: 10.3390/plants12061396 (PMC10057619; doi:10.3390/plants12061396)
Supplement: Supplementary file 1 [file plants-12-01396-s001.zip › plants-2231616-SI.pdf]

Table S1. Summary of DAS – ELISA results for different bitter gourd hybrid seeds /Variety collected from market and infected field

Sample. No	Hybrid/Variety	No of samples positive for DAS - ELISA/Total no of samples			*Percentage of embryo infection (%)
		SC	ED	EY	
**Market seeds**					
1	H1	7/30	9/30	8/30	26.6
2	H2	10/27	14/27	17/27	62.96
3	H3	7/30	10/30	6/30	20
4	H4	1/30	3/30	3/30	10
5	Variety 1	2/30	6/30	0/30	0
**Seeds from infected field**					
6	H1	5/15	7/15	4/15	26.6
7	H2	6/15	4/15	5/15	33.33
8	H3	4/15	6/15	3/15	20

***Computed on basis of embryo infection**
SC-Seed Coat, ED-Endosperm, EY-Embryo

Figure S1 (a). Begomovirus infection of H1 seeds collected from market and infected field in microplot I. (b).Mosaic. (c) Green mottling

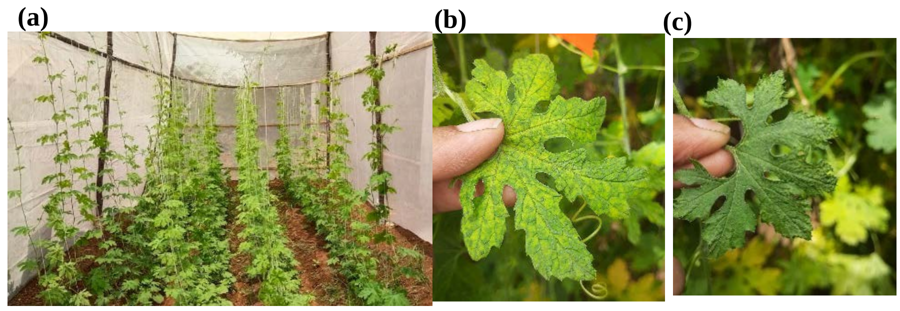

Figure S2 (a-g) Agarose gel electrophoresis of PCR products of grow-out test plants of H1 (market seeds) in microplot using ToLCNDV primer

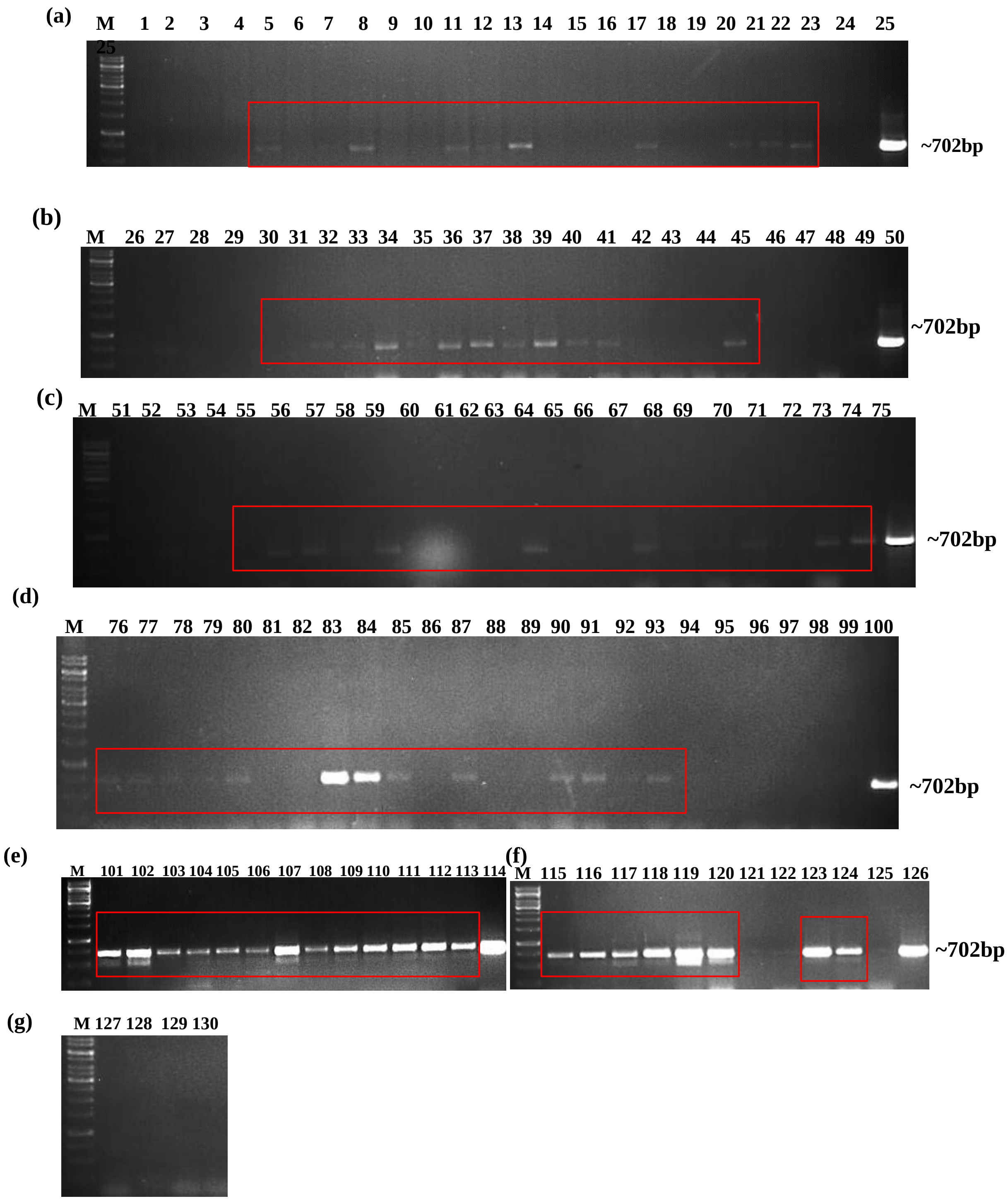

a.Lane M –1Kb Ladder, L1 – L24, b.L26 – L49, c.L51 – L74, d. L76 – L99, e. L101 – L113, f. L115 – L125, g.L127 – L130 -leaves of grow-out test plants ; L25, 50, 75,100, 114, 121, 114, 126 - Pc (infected BG leaf sample)

Figure S3 (a-f) Agarose gel electrophoresis of PCR products of grow-out test plants of H1 (market seeds) in microplot using BgYMV primer

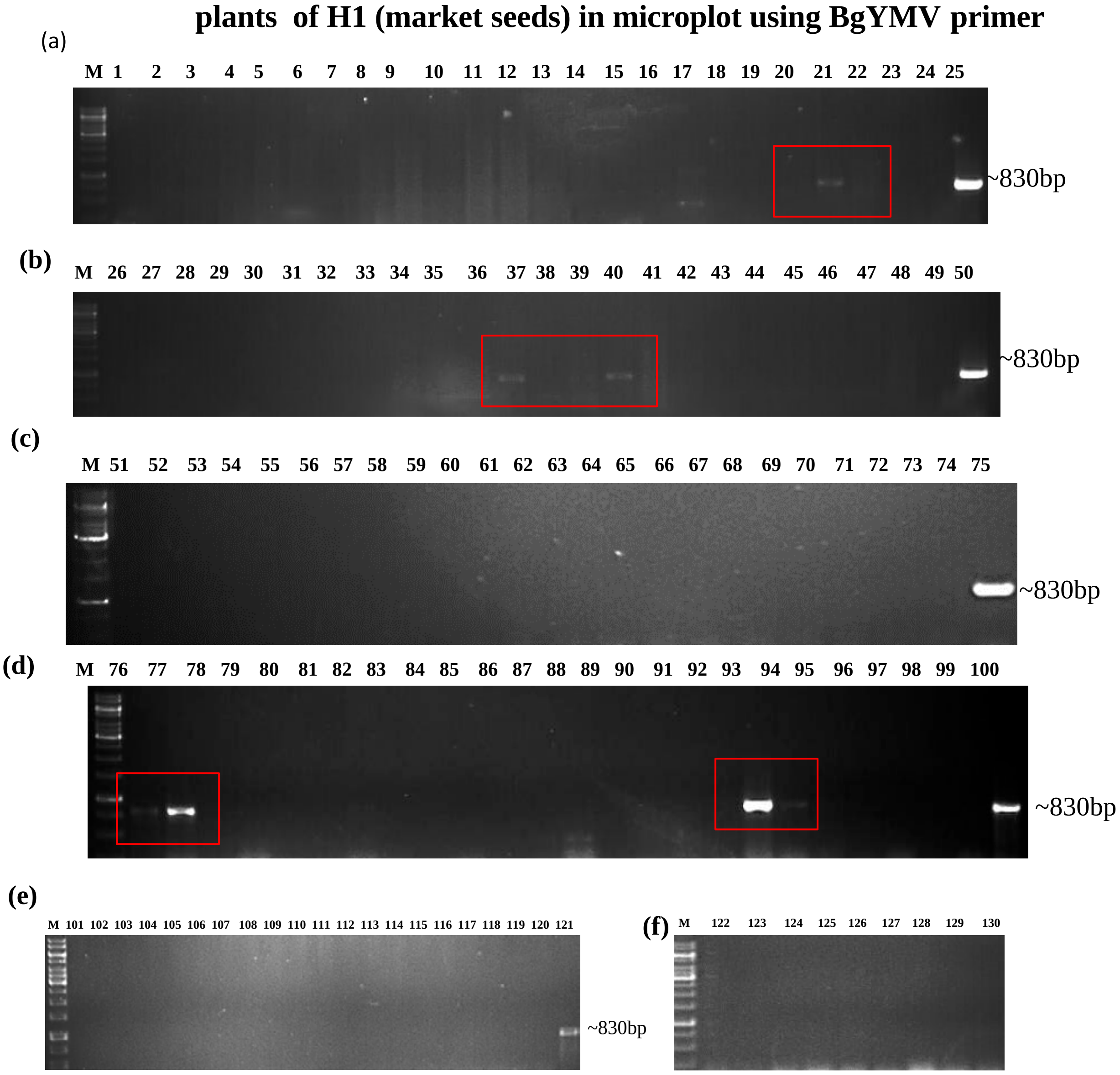

Lane M –1Kb Ladder, a. L1 – L24, b. L26 – L49, c. L51 – L74, d. L76 – L99, e. L101 – 120, f-L122 - 130 leaves of grow-out test plants; L25, 50, 75,100, 121 – PC (Infected BG leaf sample)

Table S2. Detection of begomoviruses in bitter gourd grow-out test plants (leaf samples) in microplot study

	Total no of plants in microplot	Detection of begomoviruses using PCR (170 leaf samples)					Percentage of seed transmission (In the presence of both viruses) (%)
		ToLCNDV alone		Percentage of seed transmission (ToLCNDV alone) (%)	Both BgYMV and ToLCNDV		
		Symptomatic	Asymptomatic		Symptomatic	Asymptomatic	
Grow-out test plants (H1) of seeds collected from market seed							
1	125	6/125	48/125	43.20	4/125	1/125	4.0
Grow-out test plants (H1) derived from infected fields							
2	45	1/45	0/45	2.22	3/45	1/45	8.88

Table S3. Tracking of begomoviruses in floral and fruit parts of microplot plants by PCR analysis

Source of flowers and fruits	No of samples positive / Total no of samples tested					
	Floral parts			Fruit parts		
	Sepals	Petals	Stamen	Outer rind	Inner rind	Whole seed
**Grow-out test plants derived from market seeds**						
**ToLCNDV**						
**Asymptomatic plants**						
	2/3	2/3	2/3	1/3	2/3	2/3
**Symptomatic plants**						
	2/3	2/3	1/3	2/3	2/3	3/3
**BgYMV**						
**Asymptomatic plants**						
	1/3	1/3	1/3	1/3	1/3	1/3
**Symptomatic plants**						
	0/3	0/3	0/3	0/3	0/3	0/3
**Grow-out test plants of seeds collected from infected field**						
**ToLCNDV**						
**Asymptomatic plants**						
	1/3	1/3	1/3	1/3	1/3	1/3
**Symptomatic plants**						
	1/3	1/3	1/3	1/3	1/3	1/3
**BgYMV**						
**Asymptomatic plants**						
	0/3	0/3	0/3	0/3	0/3	0/3
**Symptomatic plants**						
	0/3	0/3	0/3	1/3	1/3	1/3

Figure S4 a , b . Tracking of begomoviruses in floral and fruit parts of grow out test plants (H1) of microplot study

(a) (b)

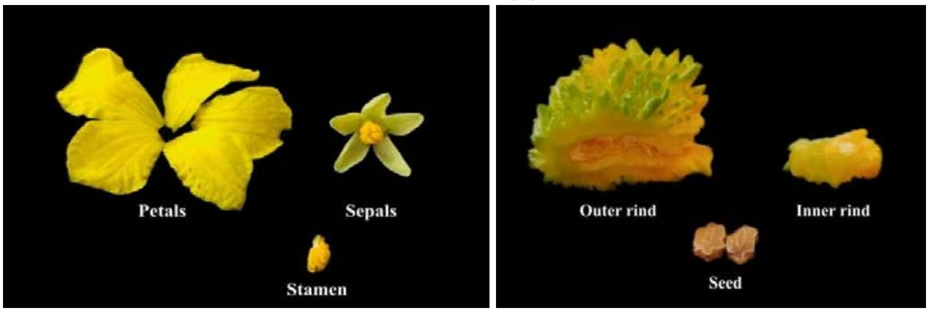

Floral parts **Fruit parts**

Figure S5 a, b. Whitefly transmission of ToLCNDV from microplot grow-out test plants

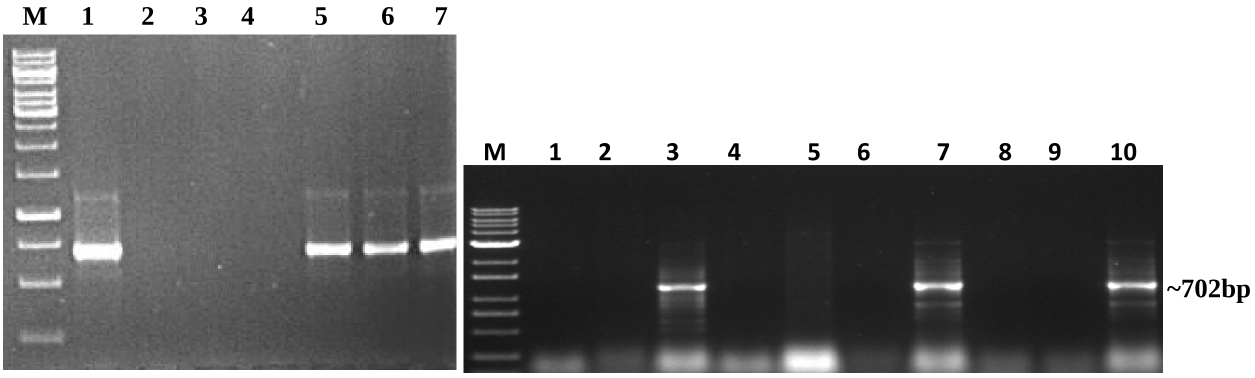

(a)Lane M –1Kb ladder, L1 – L6 leaves of whitefly transmitted plants from asymptomatic grow out test plants. L7 - PC (Infected BG leaf sample)

(b) Lane M –1Kb ladder, L1 – L9 leaves of whitefly transmitted plants from symptomatic grow out test plants. L10 - PC (Infected BG leaf sample)
